# The Use of Antioxidants as Potential Co-Adjuvants to Treat Chronic Chagas Disease

**DOI:** 10.3390/antiox10071022

**Published:** 2021-06-25

**Authors:** Edio Maldonado, Diego A. Rojas, Fabiola Urbina, Aldo Solari

**Affiliations:** 1 Programa de Biología Celular y Molecular, Instituto de Ciencias Biomédicas (ICBM), Facultad de Medicina, Universidad de Chile, Santiago 8380000, Chile; fabiola.urbina1516@gmail.com; 2 Instituto de Ciencias Biomédicas (ICB), Facultad de Ciencias de la Salud, Universidad Autónoma de Chile, Santiago 8380453, Chile; diego.rojas@uautonoma.cl

**Keywords:** *Trypanosoma cruzi*, ROS, Chagas, antioxidants, oxidative stress, mitochondrial biogenesis

## Abstract

Chagas disease is a neglected tropical disease caused by the flagellated protozoa *Trypanosome cruzi*. This illness affects to almost 8–12 million people worldwide, however, is endemic to Latin American countries. It is mainly vectorially transmitted by insects of the Triatominae family, although other transmission routes also exist. *T. cruzi*-infected cardiomyocytes at the chronic stage of the disease display severe mitochondrial dysfunction and high ROS production, leading to chronic myocardial inflammation and heart failure. Under cellular stress, cells usually can launch mitochondrial biogenesis in order to restore energy loss. Key players to begin mitochondrial biogenesis are the PGC-1 (PPARγ coactivator 1) family of transcriptional coactivators, which are activated in response to several stimuli, either by deacetylation or dephosphorylation, and in turn can serve as coactivators for the NRF (nuclear respiratory factor) family of transcription factors. The NRF family of transcriptional activators, namely NRF1 and NRF2, can activate gene expression of oxidative phosphorylation (OXPHOS) components, mitochondrial transcriptional factor (Tfam) and nuclear encoded mitochondrial proteins, leading to mitochondrial biogenesis. On the other hand, NRF2 can activate gene expression of antioxidant enzymes in response to antioxidants, oxidants, electrophile compounds, pharmaceutical and dietary compounds in a mechanism dependent on KEAP1 (Kelch-like ECH-associated protein 1). Since a definitive cure to treat Chagas disease has not been found yet; the use of antioxidants a co-adjuvant therapy has been proposed in an effort to improve mitochondrial functions, biogenesis, and the antioxidant defenses response. Those antioxidants could activate different pathways to begin mitochondrial biogenesis and/or cytoprotective antioxidant defenses. In this review we discuss the main mechanisms of mitochondrial biogenesis and the NRF2-KEAP1 activation pathway. We also reviewed the antioxidants used as co-adjuvant therapy to treat experimental Chagas disease and their action mechanisms and finish with the discussion of antioxidant therapy used in Chagas disease patients.

## 1. Introduction

Chagas disease is an illness caused by the flagellated protozoa *Trypanosoma cruzi* and it is endemic to Mexico, Central and South America, however, it has been also detected in United States, Canada, Europe, and in Western Pacific areas, due to continuous migration of people from Latin America to other countries. This disease affects 6–10 million people in endemic areas, although at least 100 million, which live in those areas, are at risk of infection by *T. cruzi*. This complex zoonotic disease causes the death of approximately 50,000 individuals each year and remains as one of the main public health problems in Latin America [1,2].

Chagas disease has two phases, which are acute and chronic, both separated by an undetermined time period in which the infected individual is relatively asymptomatic. The acute phase extends for 40–60 days with atypical and mild symptoms, therefore, misleading the early clinical diagnosis and treatment [3]. About 30% of the infected patients will develop a symptomatic chronic phase, which is characterized by either cardiac, digestive, neurological or endocrine problems [3,4]. The cardiomyopathy is the most important and severe result of Chagas disease, which leads to left ventricular systolic dysfunction, heart failure and sudden cardiac death. Most of deaths are due to heart failure (70%) and sudden death (30%) resulting from the cardiomyopathy [4]. Another manifestation of chronic Chagas disease is alterations of the gastrointestinal tract (megaesophagus and megacolon), which is related to the denervation of the enteric nervous system [2].

Chagas disease etiologic agent has a very complex lifecycle and is transmitted by several hematophagous insect vectors of the Hemiptera order, belonging to the Triatominae subfamily, which can infect more than 70 genera of mammalian hosts including wild and domestic mammals which serve as reservoirs [5,6]. Besides vectorial transmission, other routes for *T. cruzi* infection are blood transfusion or organ transplantation from infected donors, maternal–fetal transmission and infection via oral route from contaminated food and beverages [6]. Programs of vector control, blood and organ screening for *T. cruzi* and improvement of population life quality in risky areas, have helped to decrease the transmission of the parasite, however, Chagas disease is still a matter of concern in Latin American countries [7,8].

In addition to the complex life cycle of *T. cruzi*, there is a great genetic variability between different parasite populations. *T. cruzi* is quite diverse with multiple genotypes and phenotypes reported in the literature. Currently, the *T. cruzi* taxon can be subdivided into seven discrete typing units (DTUs) from TcI to TcVI and an additional TcB, which is a clade associated with bats [9,10]. The clinical outcome of Chagas disease is dependent on different factors from the parasite and host. Different DTUs can have distinct virulence factors and they have been associated with different clinical outcomes in animal models and in Chagasic patients [11]. Another important factor from the parasite is the dormancy, since dormant amastigotes are intracellular infective forms and could be resistant to drug treatment over a long period and can re-establish infection after the drug treatment [12]. Most likely, the high resistance of the amastigotes to drug exposure is dependent on the genetic background of the parasite. On the other hand, host factors, such as the genetic background, role of the immune system, age and presence of chronic diseases can have an important influence on the progression and clinical outcome of Chagas disease.

**Figure 1:**
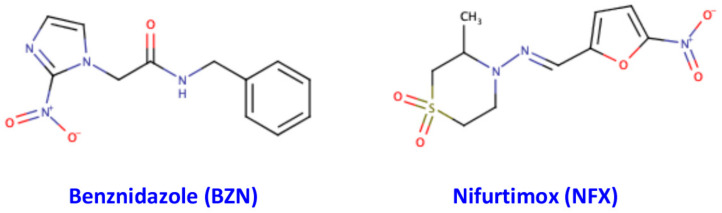
Chemical structures of Benznidazole (BZN) and Nifurtimox (NFX). Benznidazole, (IUPAC name: N-benzyl-2-(2-nitro-1H-imidazol-1-yl)acetamide) and Nifurtimox (IUPAC name: 3-methyl-4-[(E)-[(5-nitrofuran-2-yl)methylidene]amino]-1lambda6-thiomorpholine-1,1-dione) are pro-drug compounds and in the cells can be activated to act as a drug, which can react with several cellular molecular targets.

Although the above-mentioned initiatives to control *T. cruzi* infection have decreased the parasite transmission, the discovery and development of novel drugs to treat efficiently chronic Chagas disease is still a constant challenge. In the past, several compounds were used as a treatment for Chagas disease, however, all of them failed to produce efficient results. From the 1970s, a new class of drugs were used to treat Chagas disease, however, only two drugs are currently available for its treatment [13]. Both drugs belong to the same class of nitroheterocycles and they are named benznidazole (BZN) and nifurtimox (NFX), and their chemical structures are shown in Figure 1. The mechanism of action of NFX is not completely understood yet, since initially it was believed that this drug acts by oxidative stress generating free radicals with trypanocide effects [14]. However, other studies have suggested that NFX acts through a type I trypanosomal nitroreductase, which reduces the drug and forms nitriles, which inhibits parasite growth [15]. On the other hand, BZN seems to act through several different mechanisms. It has been suggested that BZN could act by a reductive stress mechanism, which involves covalent modifications of proteins, lipids and DNA of the parasite [14]. Another mechanism of action could be the reduction of BZN by a parasite type I nitroreductase, followed by several reactions resulting in the formation of dialdehyde glyoxal, which has trypanocide effect, since it can form adducts with guanosine bases of DNA and RNA of the parasite [16]. BNZ can also acts by increasing the lysis and phagocytosis of the parasite by cells of the host immune system and inhibits the growth of the parasite by the action of the fumarate reductase-NADPH enzyme [16].

NFX has been progressively discontinued due to its high toxicity and its commercialization has been suspended in Chile, Argentina, Uruguay, and Brazil [17]. However, its use still remains as an option when treatment with BZN fails but requires authorization from the PAHO (Panamerican Health Organization) or WHO (World Health Organization) for its clinical use. The major side effects of NFX are episodes of neurological disorders in the treated patients. Additionally, resistance to NFX has been reported and it is associated to the loss of a single copy of the gene TcNTR, which encodes a NADH-dependent type I nitroreductase, which is located at the mitochondrion of *T. cruzi* and it is activated by NFX to produce cytotoxic compounds against the parasite [18]. The reduction of the enzyme activity confers drug-resistance to the parasite. On the other hand, BZN is also very toxic, since it can cause several side effects, including nausea, rash, epigastric pain, abdominal swelling, nervous system disorders, white cell disorders, and eosinophilia. Additionally, there are a high number of BZN-resistant *T. cruzi* strains, which are not the result of the BZN treatment, but rather a feature of the *T. cruzi* strains and ATP-binding cassette (ABC) transporters are involved in the resistance process, since *T. cruzi* strains overexpressing the TcABCG1 gene product are naturally resistant to BZN [19,20]. Therefore, the high toxicity, parasite-resistance, and limited efficacy at the chronic phase of the Chagas disease are the main limitations for the clinical use of BZN. However, it has been shown that BZN treatment at the asymptomatic and acute phases is beneficial for the patients and BZN can also delay the progression of the disease [21,22,23]. Currently, there are no available drugs to specifically treat the chronic phase of Chagas disease. Recently, the clinical trial “Benznidazole Evaluation for Interrupting Trypanosomiasis” (BENEFIT) has demonstrated that the use of BZN does not improve the cardiomyopathy in patients with established Chagas disease at the chronic phase, despite a reduction of the parasite levels, indicating that cardiomyopathy at this stage is independent of the parasite presence [24,25]. This further confirms the need for the discovery of new drugs or combinations of them to treat efficiently Chagas disease, especially at the chronic stage. Extremely important are drugs to halt the clinical progression of Chagas disease and can also preserve and improve heart functions, since cardiomyopathy is one of the main complications at the chronic stage of Chagas disease.

Oxygen is an essential molecule for life, including human life, and the cells can be damaged when certain biochemical processes become altered and produce an excess of oxygen free radicals. Those free radicals can generate oxidative stress, which can cause damage to the cells. Free oxygen radicals are involved in the onset of many diseases including cancer, diabetes, arteriosclerosis, cardiovascular-related diseases, neurodegenerative diseases, and chronic Chagas disease. The antioxidants can function as protective agents against oxygen free radicals, thus preventing some types of cell and tissue damage. An imbalance in the antioxidant/oxidant status is considered one of the main factors associated with chronic Chagas disease progression. Macrophages and other non-immune cells, such as the cardiomyocytes, can produce elevated reactive oxygen species (ROS), which can exhaust the antioxidant defenses of the host cells and produce mitochondrial dysfunction and tissue damage. Therefore, the antioxidant therapy could be beneficial to fight the oxidative stress produced by reactive ROS and halt or lower the tissue oxidative damage produced during progression of chronic Chagas disease. In this review we will discuss the evidence regarding the use of antioxidants as co-adjuvant therapy to control oxidative stress and treat chronic Chagas disease. We will also discuss the action mechanisms of those compounds and their potential benefits.

## 2. Mitochondrial Biogenesis under Cellular Stress Response

The eukaryotic cells in response to energy demands, either by developmental signals or in response to cellular stressors, begin a mitochondrial biogenesis process, which is a self-renewed pathway in which new mitochondria are produced from the ones already existing. Many stressors induce elevated ROS production by the respiratory chain and the mitochondrion is a major ROS source (mtROS), which can produce mitochondrial injury. Cardiomyocytes produce increased ROS in response to *T. cruzi* infection, in addition to cytokines and inflammatory infiltrates in the heart. The high ROS levels produce DNA adducts, lipid peroxidation and protein damage in the myocardium in chronically infected mice and human Chagasic patients [26,27]. The mitochondrial DNA (mtDNA) damage impairs mtDNA replication and mtDNA-encoded gene transcription of the oxidative phosphorylation (OXPHOS) pathway, which leads to mitochondrial dysfunction and also affects mitochondrial biogenesis [27,28]. Therefore, the understanding of mitochondrial biogenesis is key to understand the mitochondrial dysfunction, which is produced upon *T. cruzi* infection, in an effort to prevent, block or attenuate the oxidative damage and also to improve mitochondrial functions or promote mitochondrial biogenesis. This is a complex and multistep process that engages coordination between the nuclear and mitochondrial genomes (bi-genomic) with a retrograde signaling that can change the global nuclear gene expression pattern. This multistep process involves (i) mtDNA replication and transcription, (ii) translation of mtDNA-encoded genes, and (iii) synthesis, import and assembly of nuclear DNA-encoded mitochondrial proteins.

Mitochondrial DNA replication is carried out by DNA polymerase γ, which is a heterotrimer with one catalytic subunit POLγA and two accessory subunits POLγB, which enhances interactions with the DNA template and stimulates both the catalytic and the processivity of POLγA [29,30]. Additional protein factors are required for mtDNA replication, such as a primase, helicase (TWINKLE) and a single-stranded DNA-binding protein (mtSSB), which protects it against nuclease attack and also stimulates the TWINKLE helicase as well as the processivity of POLγ [30]. Mitochondrial DNA has a limited coding capacity and requires that most of the mitochondrial proteins necessary for respiratory, oxidative and biosynthetic pathways have to be nuclear-encoded and then imported into the mitochondrion. The mtDNA encodes only 13 essential proteins, which functions as essential subunits of the OXPHOS system (complexes I, III, IV, and V). Those genes are transcribed in a bidirectional form in multigenic transcripts, which are processed, polyadenylated and translated inside the mitochondrion [30]. Two mitochondrial ribosomal RNAs and 22 mitochondrial transfer RNAs are also encoded by the mtDNA [31]. The core machinery of mitochondrial gene expression is based on a single mitochondrial RNA polymerase (POLRMT), the initiation (TFB1M and TFB2M) and stimulatory (Tfam) transcription factors and a family of transcription termination factors (mTERFs) [31].

**Figure 2:**
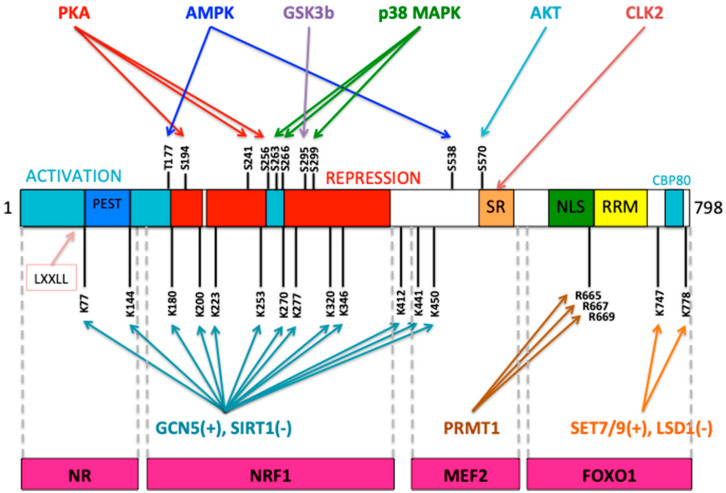
Domain structure and post-translational modifications of PGC-1α coactivator. This transcriptional coactivator possesses different domains to achieve its function. Mainly, the N-terminal possesses the activation domain, the middle contains the repression domain and the C-terminal contains several regulatory domains as is shown in the figure. The LXXLL motif is located at the N-terminal into the activation domain and most of the regulatory post-translational modifications are grouped at the N-terminus, mainly phosphorylation sites and modified lysines, which can be acetylated by GCN5 or deacetylated by SIRT1 and SIRT3. Arginine at the C-terminus can be methylated by PRMT1 (Protein arginine methyltransferase 1). SR (serine-arginine-rich domain), NLS (nuclear localization sequence), RRM (RNA recognition motif), CBP80-binding domain. On the bottom are indicated the regions in PGC-1α, which can interact with transcription factors such as nuclear receptors (NR), nuclear respiratory factor 1 (NRF1), myocyte enhancer factor 2 (MEF2) and forkhead box O1 (FOXO1). This figure was reproduced and modified from Miller et al. 2019 [38].

Mitochondrial biogenesis and respiratory function are mainly governed by a transcriptional control. The peroxisome proliferator-activated receptor gamma (PPARγ) coactivator-1 family (PGC-1) of transcriptional coactivators, which consists of PGC-1α, PGC-1β, and PRC (PGC related coactivator) are the key regulators of mitochondrial biogenesis [32,33]. Those transcriptional coactivators are inducible regulators of nuclear receptors (transcription factors) and transcription factors (transcriptional activators), which can regulate gene expression and control cellular energy metabolism process. PGC-1α is a master regulator of mitochondrial biogenesis and it is highly expressed under high cellular energy demands, for example, exercise, cold, fasting, cancer progression, neurodegenerative diseases, pathological states and especially in tissues with high energy demand [33]. This transcriptional coactivator plays important roles in the expression of mtDNA and nuclear-encoded genes that drive mitochondrial biogenesis and increase the mitochondrial OXPHOS function [32,33]. The mitochondrial biogenesis process is initiated by the activation of PGC-1α, either by deacetylation (via SIRT1) or phosphorylation (through AMPK or p38MAPK and deactivation by protein kinase B PKB/AKT) [33,34], which in turn stimulates nuclear transcription factors, such as nuclear respiratory factors 1 and 2 (NRF1 and NRF2) which regulate the expression of Tfam, an essential nuclear-encoded transcription factor for maintenance, replication and transcription of mtDNA [35,36]. PGC-1α activity can be regulated through an inhibitory acetylation by GCN5 or by methylation by protein arginine methyl transferase (PRMT1) [33,34,37,38]. Energy metabolism is affected by PGC-1α status through its interactions with transcription factors NRF1, NRF2, YY1, ERRα and others involved in the transcriptional control of nuclear-encoded genes involved in mitochondrial biogenesis or OXPHOS activity [32,38]. PGC-1 family of proteins can interact with different transcription factors and with nuclear receptors (NR) through the LXXLL motif (NR-boxes), present in the N-terminal domain and this motif is a coactivator signature that enables the PGC-1 polypeptides to interact with nuclear receptors to exert their transcriptional activity on target genes [38]. The domain structure of PGC-1α and the function of each domain is displayed in Figure 2. Additionally, important residues that can be modified are shown in Figure 2.

Cytochrome c oxidase (COX) is one of the mitochondrial bi-genomic encoded complexes having 10 subunits nuclear-encoded and three mitochondrial-encoded. All 10 nuclear-encoded subunits contain in their gene promoters NRF1 binding sites and their gene expression is regulated by NRF1 in neurons [39]. NRF2 also plays a role controlling the transcription of all 10 nuclear-encoded COX subunits in neurons [40,41], furthermore, it controls the transcription of the cytochrome c encoding gene and many nuclear-encoded genes involved in the expression and function of the electron respiratory chain [33]. PGC-1α also functions as a transcriptional coactivator for transcription factors involved in growth or stress signaling, such as p53, HSF1 (heat shock factor 1) and YY1 [42,43,44]. Recently, it has been shown that heat shock produces perinuclear accumulation of mitochondria in mammalian cells, which can induce increased nuclear ROS production, which leads to activation of hypoxia-inducible factor 1α (HIF-1α), which is essential for HSF activation and HSF1-mediated heat shock response [45].

Although the mitochondrial biogenesis process is a normal response to the energy demand triggered by proliferation signals, it also acts in response to cellular stressors, such as caloric restriction and then this process is increased in order to stimulate mitochondrial energy metabolism. Another cellular stressor to respond is the elevated ROS level, which are generated as natural by-products under a high respiratory condition. In such cases, PGE-1α is activated to launch mitochondrial biogenesis to compensate the mitochondrial damage produced by respiratory chain defects.

Mitochondria are critical organelles acting as cellular hubs, in which catabolic and anabolic cellular processes can converge and integrate. PGC-1α is a master regulator controlling mitochondrial biogenesis and oxidative functions to maintain the energy homeostasis in response to cell signals. There are at least five different mitochondrial dysfunctions which leads to the generation of retrograde signals, namely, (i) AMP/ATP ratio, (ii) NAD^+^/NADH ratio, (iii) ROS production, (iv) Cytosolic Ca^+2^ signals, and (v) Mitochondrial membrane potential reduction.

In response to energy stress, AMP activates AMP protein kinase (AMPK; AMP-activated protein kinase), which is the main regulator of mitochondrial biogenesis under acute energy crisis and is an energy sensor of the cell. AMPK exerts its function by activating SIRT1, a PGC-1α activating deacetylase, which in turn can serve as coactivator for NRF1 and NRF2 transcription factors, thereby activating mitochondrial biogenesis and OXPHOS [34,46]. The mammalian target of rapamycin (mTOR), a large protein kinase, can play an opposite role to AMPK, since mTOR is able to stimulate anabolic pathways under high nutrient conditions [47]. These opposite functions could reflect that AMPK and mTOR evolved as a Yin-Yang-like antagonistic mechanism to control catabolism and anabolism. YY1 binding motif (ATGGCG) is enriched upstream of promoters of predicted nuclear-encoded mitochondrial genes and is highly present upstream of promoters of nuclear-encoded oxidative phosphorylation genes, indicating that YY1 might regulate their expression [44]. Indeed, YY1 controls the expression of those genes through mTOR, which is able to interact with YY1. Furthermore, YY1 is also found in the promoter regions of PGC-1α encoding genes, although it seems that it does not bind directly, but instead through the interaction with the raptor factor, which is a component of mTORC1 [44]. This is a mechanism by which mTOR balances energy metabolism by using the transcriptional control of mitochondrial oxidative functions. Moreover, transcription factor YY1 can regulate, in either positive or negative control, the expression of certain COX subunit encoding genes [33].

Transcription factor CREB (cAMP Response Element-Binding) is also necessary for cAMP-dependent gene expression of cytochrome c, since the promoter region contains two cAMP response elements (CREs), which can bind the CREB protein, leading to the activation of cytochrome c gene expression [48]. The expression of the cytochrome c gene is induced by cAMP through a mechanism involving transcriptional activation PKC/CREB pathway. Those observations indicate that respiratory chain gene expression can be regulated by cAMP by means of a CREB-dependent signal transduction pathway [48,49]. Transcription factor CREB is able to locate at the mitochondria and gets imported into the mitochondria through a TOM (translocase of the outer membrane) complex with the aid of a mitochondrial heat shock protein 70 (mtHSP70) [50]. Chromatin immunoprecipitation indicated that CREB binds to the CREs present in the D-loop of mtDNA and its depletion within the mitochondria interferes with the expression of several mitochondria-encoded mRNAs of complex I, thus decreasing complex I activity [50,51]. On the other hand, PGC-1α gene expression depends on CRE, since its promoter contains CRE binding sites (TGACGTCA), which enables PGC-1α gene expression and response to cell signals such as cAMP and calcium signaling [52].

**Figure 3:**
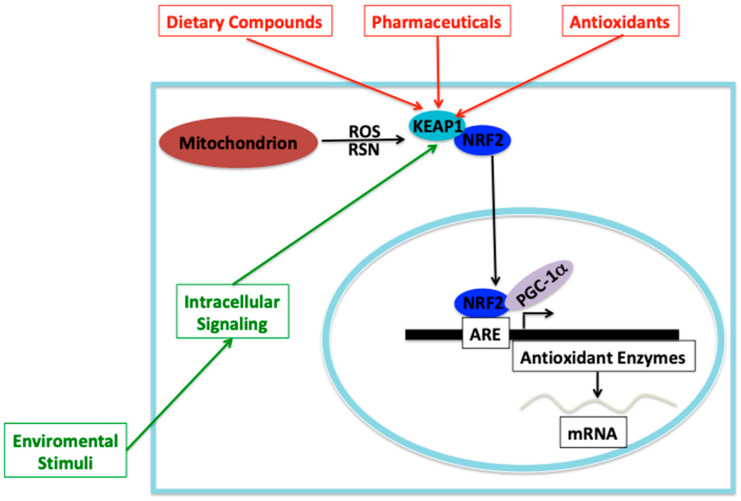
Signaling pathways and mitochondrial biogenesis. There are multiple signaling pathways that could lead to mitochondrial biogenesis. The main cellular energy sensor is the AMPK, which is able to sense the low energy status and transduces the signal by phosphorylating SIRT1, which in turn can deacetylase PGC-1α to activate it to perform transcriptional coactivation on the NRF transcriptional (NRF1 and NRF2) activators to stimulate gene expression of mitochondrial genes to start mitochondrial biogenesis. Furthermore, AMPK can directly activate PGC-1α via phosphorylation. Additionally, certain stimuli can elicit calcium signaling to activate CaMK, which activates p38MAPK to phosphorylate PGC-1α to activate its transcriptional coactivator function and launch mitochondrial biogenesis. Environmental stimuli can directly activate CaMK, which can signal p38MAPK to phosphorylate PGC-1α and activate it. On the other hand, environmental stimuli or cAMP can activate PKC, which is able to phosphorylate CREB, which in turn can bind to the CRE on the gene promoter of PGC-1α gene to augment its expression, which can be activated by the mentioned signals to begin the mitochondrial biogenesis process. All signals can augment the gene expression of nuclear-encoded mitochondrial proteins involved in OXPHOS function, Tfam mitochondrial transcription factor and mitochondrial structural proteins.

Calcium signaling is involved in mitochondrial biogenesis through protein kinases, PGC-1α and CREB transcription factor. Elevation of intracellular calcium is able to activate cytoplasmatic protein kinases such as PKC and calcium/calmodulin-dependent protein kinase (CaMK), which can activate the gene expression of several nuclear-encoded genes necessary for mitochondrial biogenesis and mitochondrial functions. CaMK in response to high calcium levels is able to activate p38MAPK, which in turn can activate PGC-1α by phosphorylation [53]. On the other hand, in response to exercise/neuromuscular activity, p38MAPK is activated, which in turn directly activates the downstream target ATF-2 (activating transcription factor 2), a member of the CREB family, which then binds to the CRE motif on the PGC-1α promoter, leading to activation of gene expression that promotes mitochondrial biogenesis and OXPHOS function [53,54]. PGC-1α is capable of coactivating the transcription factors NRF1 and NRF2, which results in the transcriptional activation of several genes involved in the transcription of mtDNA-encoded genes, nuclear-encoded OXPHOS subunits and nuclear-encoded mitochondrial protein importers, all of those involved in mitochondrial biogenesis [53]. Mitochondrial ROS production can launch mitochondrial biogenesis through PGC-1α induction [55], unfortunately the published data are contradictory and the mechanism of PGC-1α induction by mtROS is completely unknown. Clearly, there is a crosstalk between mtROS production and mitochondrial biogenesis in mammalian cells, however, there are few studies and the data is conflictive, therefore, we cannot discuss this issue further. Mitochondrial biogenesis can also be stimulated by stimuli, such as nitric oxide and hypoxia and other injuries that can cause mitochondrial disorders [56]. The main signal transduction pathways leading to mitochondrial biogenesis are shown in Figure 3.

Mitochondrial biogenesis in the heart is worth mentioning, since one of purposes of the antioxidant therapy for Chagas disease is the stimulation of the mitochondrial biogenesis and the improvement of the OXPHOS function. The heart requires a high mitochondrial biogenesis capacity in order to support the large energy demand for ATP production from fatty acids as the predominant fuel substrate. Key proteins involved in mitochondrial biogenesis in the heart are PGC-1 polypeptides family and the importance of these transcriptional coactivators has been studied by gene deletion in mice. Cardiac-specific transgenic overexpression of PGC-1α in mice can result in a large mitochondrial biogenic response during the postnatal period and also an increased expression of nuclear-encoded mitochondrial genes [57]. Deletion of PGC-1α or PGC-1β does not lead to abnormalities in mitochondrial biogenesis or OXPHOS function under normal conditions, indicating that PGC-1α and PGC-1β play a redundant role. However, the loss of any of them accelerates mitochondrial dysfunction under stress of pressure overload [58]. The germline deletion of both PGC-1α and PGC-1β produces a perinatal heart failure, which is lethal, and it is caused by a total lack of mitochondrial biogenesis [59]. PGC-1 induced deletion in adult mice does not alter mitochondrial density, however, produces a subset of mitochondria with collapsed cristae reminiscent of the pathological phospholipid seen in the human Barth syndrome, a congenital disease caused by altered cardiolipin biosynthesis [60]. Those observations highlight the fundamental role of the PGC-1 family in heart function. Another transcription factor that contributes to mitochondrial biogenesis is c-Myc that induces gene expression of NRF1 and Tfam [61,62]. Interestingly, c-Myc can trigger increased glucose use instead of fatty acids during heart growth or ischemia accidents, suggesting that c-Myc plays a role in mitochondrial biogenesis, when canonical pathways are inactive, such as during the fetal period, when the heart relies on glucose as the principal source of fuel [61,62].

In vertebrate organisms, the molecular mechanisms by which extracellular signals regulate mitochondrial function and biogenesis are still largely unknown. The studies show that there is an intricate complex mechanism, which involves multiple cell signals and transcriptional factors. The master regulator PGC-1α which is a coactivator for nuclear receptors and different transcription factors, can regulate gene expression of several mitochondrial components. The knowledge of the signals and components is crucial to understand the metabolic diseases, neurodegenerative diseases, aging, cancer and pathological states, such as Chagas disease.

## 3. NRF2-KEAP1 Signaling under Oxidative Stress

**Figure 4:**
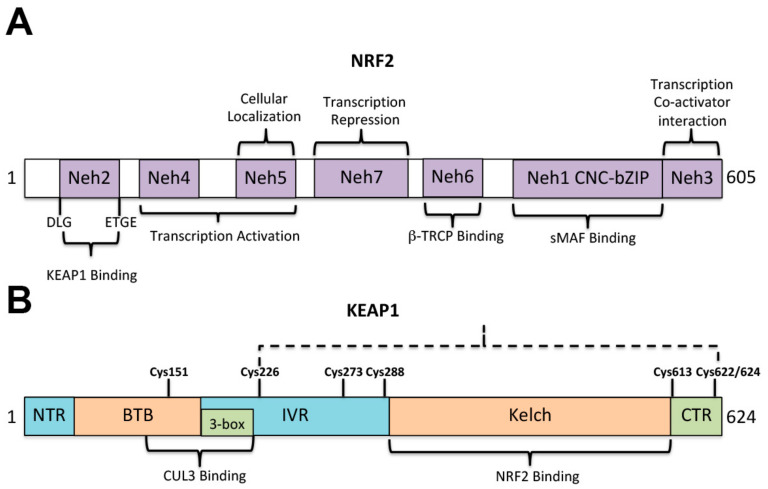
The main functional domains of NRF2 and KEAP1 polypeptides. (**A**) NRF2 possesses seven functional (Neh1-7) domains that are involved in the regulation of its transcriptional activity and stability. The KEAP1 binding domain is located at the Neh2 domain, β-TRCP (beta transducin repeat containing protein) binding is located at the Neh6 domain and sMAF (small musculoaponeurotic fibrosarcoma) transcription factor binding is located at the Neh1 domain. The ARE binding domain is located at the CNC-bZIP (Cap and collar basic leucine zipper) Nhe1 domain. The main relevant domain functions are indicated over the figure. (**B**) KEAP1 possesses five domains including NTR (N-terminal region), BTB (Bric a brac, tramtrack and broad complex), IVR (intervening region), Kelch (Kelch or DGR domains, two Glycine repeat domains responsible for NRF2 binding), and CTR (C-terminal region). The binding domain for CUL3 comprises regions of BTB and IVR, while the binding domain for NRF2 is in the Kelch region. The stress sensors are shown by a dashed line, which links the three parts of the H_2_O_2_ stress sensor center. KEAP1 relevant domain functions are indicated over the figure. This figure was reproduced and modified from Baird and Yamamoto 2020 [65].

**Figure 5:**
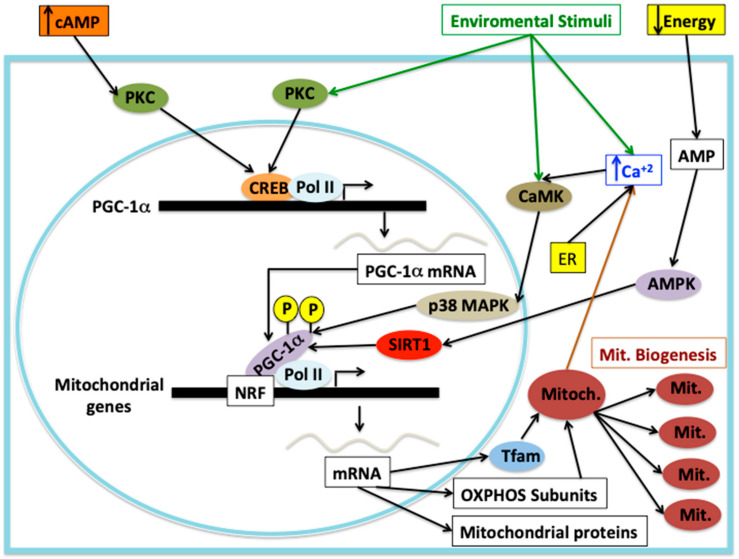
The ARE-NRF2-KEAP1 signaling pathway. Several factors (phase II inducers) can activate the ARE-NRF2-KEAP1 pathway to trigger antioxidant enzymes expression (phase II gene products). The pathway starts with the signaling from phase II inducers, such as pharmaceuticals, oxidants, antioxidants, electrophile compounds, dietary compounds, and intracellular signals which can be sensed by cysteine residues on the KEAP1 polypeptide, which release NRF2 to be translocated to the nucleus and bind to the ARE sequences in the gene promoter of antioxidant enzyme encoding genes to start the transcriptional activation of those genes and mount a cellular antioxidant defense response. Mitochondrial ROS/RSN or hydrogen peroxide can also signal on KEAP1 to release NRF2 and start the cellular antioxidant defense.

The nuclear transcription factor erythroid 2-like 2 (NFE2L2), also named NRF2, in conjunction with the cytosolic Kelch-like ECH-associated protein 1 (KEAP1) system, is a defense system developed to preserve the cellular homeostasis against the oxidative and electrophilic stress. NRF2 was first described as a transcriptional activator of the β globin gene cluster [63] (Figure 4A,B). Under normal conditions (homeostasis) NRF2 is associated with KEAP1 into the cytoplasm and is ubiquitinated (by a E3 ubiquitin ligase) to be targeted for proteosome-dependent degradation [64,65]. NRF2-ubiquitination and subsequent proteasome degradation keeps the NRF2 activity at very low levels under normal conditions. KEAP1 is a cysteine-rich protein, which has five functional domains and one of those domains (IVR) can interact with E3 ubiquitin ligase (CUL3), which marks NRF2 for proteosome-dependent degradation [64,65] (Figure 4A). Under oxidative stress conditions, modification of cysteine sensors in KEAP1 facilitates NRF2 to lose its interaction with KEAP1, escaping from ubiquitination to be accumulated into the cell and then translocate to the nucleus through its Neh1 domain (Figure 4A) to bind the antioxidant responsive element (ARE) sequences present in the promoter of target genes to activate the expression of antioxidant and metabolic mitochondrial genes [65,66,67]. The Nhe1 domain contains a CNC-bZIP domain which can bind to the ARE element (Figure 4A). The ARE element is also known as electrophile response element (EpRE) and has a consensus sequence TGACNNNGC. In this way, and in response to oxidative or electrophilic stress, NRF2 can upregulate the gene expression of the called phase II gene products, which are able to exert cyto-protection, detoxify, including glutathione synthesis, ROS scavenging, detoxification, drug metabolism, and transport [64,68,69,70,71]. Inducers of oxidative stress and phase II enzymes are structurally diverse, however, they all have a common property that lead Talalay et al. [66] to propose a mechanism by which the cysteine thiols of KEAP1 reacts with the electrophilic center of the phase II inducer disrupting the NRF2-KEAP1 complexes [65,66], allowing NRF2 to translocate to the nucleus to bind the ARE elements and activate gene expression of phase II cytoprotective enzymes (Figure 5). Although, KEAP1 is a cysteine-rich protein, a few key cysteines are able to act as damage sensors and those are shown in Figure 4B.

ROS is a phase II inducer, which is produced by *T. cruzi*-infected macrophages and cardiomyocyte mitochondria. Recent results indicate that the redox state of *T. cruzi* is key for cell growth and a signaling pathway responsible for transducing the signal might exist, however, this signal is still unknown. Proliferation of *T. cruzi* epimastigotes treated with antioxidants, oxidants and prooxidants has been evaluated and while antioxidants were able to reduce proliferation and increase the differentiation to metacyclic trypomastigotes, a form which does not proliferate, the oxidants and prooxidants had the opposite effect [72]. Some authors have proposed that H_2_O_2_ could be the signaling molecule, due its high diffusional capacity to cross biological membranes [73]. Those authors have proposed that ROS might play a dual role in the *T. cruzi* life cycle, in which high ROS levels are necessary for cell proliferation and low ROS levels (reduced environment) are obligatory to promote differentiation (metacyclogenesis), a process that occurs naturally inside the insect vector hindgut under nutritional stress conditions and plenty of heme, as a side product of blood digestion [74,75].

ROS produced in *T. cruzi*-infected cells should elicit an antioxidant defense, most likely mainly through the NRF2-KEAP1 pathway, however, the persisting high ROS levels exhaust the antioxidant defenses leading to mitochondrial dysfunction, impaired mitochondrial biogenesis, cardiomyopathy and fibrosis, which are characteristic of chronic Chagas disease [76,77,78]. Indeed, several studies have shown that the mitochondrial host is the main source of ROS (mtROS) in *T. cruzi*-infected cardiomyocytes and in Chagas disease patient hearts [78,79]. An unbalance of antioxidant/oxidant is considered a major factor associated with Chagas disease progression [80,81]. In the myocardium of chronically infected animals and Chagasic patients several enzymatic and non-enzymatic antioxidant systems exist to control the oxidative stress [80,81], however, they might get exhausted due to the high mtROS production.

In a recent study of *T. cruzi*-infected mice at the chronic phase of Chagas disease, it was observed a myocardial loss of mitochondrial membrane potential, loss of complex II-driven respiration and 9-fold increase in mtROS production [77]. Both, in vivo and in vitro studies demonstrated that *T. cruzi* infection resulted in ROS-dependent decline in the associated activities of NRF2 [77], leading to the decrease of the antioxidant response mediated by the ARE response element, which resulted in a large decline (35–99%) in antioxidant enzymes expression, such as heme oxygenase-1 (HO-1), glutathione S transferase (GST), thioredoxin (Tr), NADPH dehydrogenase, and several others. There was also an increase in myocardial/mitochondrial oxidative stress adducts, left ventricular (LV) mass and high collagen synthesis in *T cruzi*-infected mice [77]. The overexpression of manganese superoxide dismutase (MnSOD) in Hela cells or cultured cardiomyocytes and in infected-MnSOD transgenic-mice was able to preserve NRF2 transcriptional activity and the antioxidant/oxidant balance, whereas cardiac oxidative damage and the fibrotic pathway were considerable reduced [77]. Importantly, mutant MnSOD^+/−^ mice, in which the expression of this enzyme is decreased, had a high mitochondrial ROS production, increased myocardial oxidative damage and mitochondrial dysfunction [82]. Those results indicate the importance of NRF2 in the antioxidative response and its inhibition by ROS can signal the fibrotic pathway and the progression of chronic cardiomyopathy. Therefore, a therapy to improve the antioxidant defenses of the host during *T. cruzi* infection is a key issue to consider. Thus, treatment with antioxidants might be fundamental to halt Chagas disease progression and improve the heart functions in chronically infected patients.

## 4. Antioxidant Drugs

Antioxidant co-adjuvant therapy could be a promising strategy to treat chronic Chagas disease, since oxidative stress contributes to disease progression and host tissue damage, particularly the heart due to the strong tropism of *T. cruzi* for the cardiomyocytes [83]. One of the main factors associated with Chagas disease progression is an imbalance of antioxidant/oxidant due mainly to the oxidative stress. Several studies in mice and humans have shown that host immune system controls the parasite infection by producing ROS/RNS species, proinflammatory cytokines, antibodies against the parasite, and cytotoxic T lymphocytes [84,85]. Natural immune cells, mainly macrophages, are the first defense line against *T. cruzi* infection and they respond through an immediate increase in the production of proinflammatory cytokines, followed by the production of superoxide by NADPH oxidase (NOX2) and nitric oxide by the inducible nitric oxide synthetase (iNOS) [85,86,87]. The reaction of superoxide and nitric oxide produces peroxynitrite, which is one of the most powerful cytotoxic agents to kill the infecting parasites [87,88], however, it can also exert cytotoxic effects on the host cells. Moreover, it has been shown that the oxidative environment produced by the macrophages acts as a positive signal for *T. cruzi* proliferation [72]. Other non-immune cells, such as cardiomyocytes, can also react to *T. cruzi* infection by producing ROS and several studies have identified their mitochondria as the ROS source [89,90]. The increased ROS production from the cardiac myocytes produces mitochondrial dysfunction, which leads to several adverse effects on the heart function. Studies in humans and rodents have demonstrated that an increased mitochondrial ROS production and mitochondrial defects persist in the chronic phase of Chagas disease [90,91].

Increased production of proinflammatory cytokines by macrophages and dendritic cells is an additional factor that can contribute to the progression of Chagas disease and ROS levels are critical for signaling the nuclear factor κB (NF-κB)-dependent expression of proinflammatory cytokines, suggesting that NOX2/ROS produced in macrophages might signal the transcription factors to promote cytokine gene expression, however, the mechanisms are not fully understood yet [78,92,93]. Hence, the persisting high levels of cytokines can influence the progression of Chagas disease towards the chronic phase.

The oxidative stress is controlled by a complicated network of antioxidant enzymatic and nonenzymatic antioxidant molecules. The balance between ROS capable to induce parasite killing and the antioxidant host machinery required to detoxify and maintain a safe environment for the host cells is essential. However, sometimes the host antioxidant response can be exhausted, and Chagas disease progresses to the chronic phase self-perpetuating mitochondrial dysfunction and mitochondrial ROS production in the cardiac myocytes, leading to a heart failure. The increased mitochondrial ROS production by the cardiomyocytes elicits an antioxidant depletion, persistent inflammation, oxidative damage of proteins, lipids, DNA and produces a fibrotic gene expression response, which is a characteristic manifestation in the heart of chronic Chagas disease patients [92,93,94,95]. Consequently, several antioxidants have been studied as complementary or co-adjuvant therapy together with antiparasitic drugs to treat the oxidative tissue damage and the cardiomyopathy produced at the chronic phase of Chagas disease. The antioxidants used in those studies included vitamin C (vitC), vitamin E (vitE) or a combination of both, melatonin, curcumin, resveratrol, astaxanthin, phenyl-a-tert-butyl nitrone, tempol, desferrioxiamine, honokiol, phytochemical compounds, apocynin, and SIRT1 agonists. The effect of antioxidants in order to control oxidative stress and improve heart function have been evaluated using murine models, which were infected with *T. cruzi*, in both the acute and chronic phases of Chagas disease. This murine model or experimental Chagas disease reproduces most of the features observed in Chagas disease patients. The acute phase was defined up to 60 days after infection and the chronic phase from day 120 after the initial infection with blood trypomastigotes. Except for studies with vitC/vitE, most of the studies have not evaluated the effect of the antioxidant on the parasite burden, rather they focused on the measure of the oxidative stress levels and the improvement of heart function. Recently, a systematic review of studies evaluating antioxidants and anti-inflammatory drug therapies carried out in Brazil on patients with Chagasic myocarditis has been published. Those studies were published from 2002 to 2017 and evaluated the use of vitC/vitE as an antioxidant therapy and evaluated also the effect of anti-inflammatory drugs. The results of those studies will be detailed later.

### 4.1. VitC/VitE

VitC is one of the most frequent antioxidants used in studies of Chagas disease and it is ascorbic acid, one of the most powerful antioxidant agents, however, it can also act as a pro-oxidant [95]. The studies have used mice, but with distinct experimental conditions such as *T. cruzi* strain, antioxidant dose, parasite load, or the treatment time period. Some of those studies evaluated the VitC effect of on the acute phase, others on the chronic phase of Chagas disease and also on both of them. The trypanocidal effect of vitC on a murine model of acute Chagas disease was similar for the three parasite forms of *T. cruzi*, however, it did not induce any change on the antiparasitic activity of BZN on *T. cruzi*, when both compounds are combined [96]. In mammal cells vitC is able to diminish the cytotoxic effects of BZN, since it acts as an antioxidant agent. Parasite load was lower in infected mice treated with vitC compared to the control group, but the mice treated with BZN had lower parasitemia levels compared to the vitC treated group [96]. VitC seems to have a lethal pro-oxidant effect on *T. cruzi* when used alone and also an antioxidant effect on the host if combined with BZN. VitC was able to decrease the cytotoxicity of BZN and the *T. cruzi* infected vitC treated mice did not exhibit weight loss and had a 100% survival during the acute phase of parasite infection [96]. In a related study [97], the use of low doses of BZN together with vitC reduced parasitemia in mice at the acute phase of Chagas disease and the combination was more effective than in mice receiving BZN or VitC alone. Additionally, the combined treatment was effective in reducing macrophage intracellular ROS production and lipid peroxidation in the heart and it was effective to reduce cardiac parasitism. Those results indicate that vitC might improve the trypanocidal activity of BZN and attenuate its toxic effects on the host. This combined treatment could lower the oxidative damage and inflammation in infected individuals, which could lead to an increased cardio-protection. On the contrary, other researchers showed that vitC treatment is unable to protect against the oxidative stress at both the acute and chronic stages of Chagas disease, rather promotes an increase of the inflammatory process and produces greater host tissue damage [98,99]. It has been noticed that vitC treatment increased the lipid peroxidation in a murine model and concluded that vitC acts as a prooxidant and proinflammatory agent. Recently, Martins et al. [100] also reported that vitC acts in a contradictory and simultaneous manner, since in determined moments of the acute phase it can act as a pro-oxidant by increasing NO concentration and decreasing glutathione (GSH) and it acts also as an antioxidant by increasing ferric-reducing ability of plasma (FRAP) and uric acid concentrations. At the chronic phase of Chagas, there was a decreasing of the FRAP and uric acid concentrations, indicating that there was a bigger use of the host antioxidant defenses against *T. cruzi* infection, indicating that the use of vitC at the utilized dose is unable to stop the oxidative stress induced by *T. cruzi* infection. The main reasons for the observed differences in the result of those related studies could be the vitamin treatment protocol and the *T. cruzi* strain used in the experiments.

In a study of *T. cruzi* infected mice they were treated with vitamins C, E or both for 60 and 120 days [101]. The results showed that the parasitemia was the same among the vitamin treated groups, however, those groups had more severe inflammation in the skeletal muscle compared with placebo treated groups in both acute and chronic phases of Chagas disease. At the acute phase, the mice displayed increased FRAP and GSH levels in both the vitC and vitC/vitE treated groups. In the chronic phase of Chagas disease, a decrease in GSH levels was observed in the vitE treated group and also a decrease was determined in thio-barbituric acid reactive substances (TBARS; lipid peroxidation) in the vitC/vitE treated group. Additionally, a decrease in TBARS in the cardiac tissues was observed in the vitC and vitC/vitE treated groups, however, the levels of TBARS were higher in the vitE treated group than in the vitC treated group. In a related study, *T. cruzi* infected mice were treated with vitC or vitE [102]. After a time period of 20 days, the mice had reduced vitC and vitE tissue levels, but high proinflammatory cytokine and prostaglandin F2α (PGF2α) levels were found. The NO cardiac production was increased and there was an intense myocarditis and reactive host tissue damage, which directly correlates with the degree of the inflammatory infiltrate and the pathological cardiac remodeling. In conclusion, this study showed that vitC and vitE could not counterattack host reactive tissue damage produced by *T. cruzi* infection at the acute phase of Chagas disease [102].

### 4.2. Melatonin

Melatonin is an indoleamine and a nightly secretory product of the pineal gland, which produces a wide range of biological responses in several tissue targets. Its action is mediated through receptor-mediated and receptor-independent mechanisms with pleiotropic functions including circadian pacemaker, antioxidant, antioxidative enzyme gene expression regulator, free radical scavenger and anti-inflammatory agent [103]. Earlier studies evaluating the role of melatonin to stimulate the immune system of rats were carried out by Santello et al. on the acute phase of Chagas disease [104]. In this study, melatonin was administered either seven days prior to the infection with *T. cruzi* or concomitant with the infection. It was shown that both treatments could increase proinflammatory cytokine levels such as IL-12, TNF-α and IFN-γ, although the concomitant treatment was more effective. NO concentrations were reduced under melatonin therapy, indicating that melatonin can reduce the oxidative stress produced in acute Chagas disease. In a study in which rats after 60 days post *T. cruzi* infection were treated with melatonin and several parameters were evaluated, melatonin treatment decreased TNF-α and NO produced by cardiomyocytes, however, increased IL-10 levels [104]. Moreover, this treatment led to decreased heart size and decreased inflammatory foci when compared to infected untreated controls. This suggests that melatonin can protect of the severe cardiomyopathy which is a feature of chronic Chagas disease. In a related study carried out by Brazao et al., melatonin was orally administered for two months during chronic Chagas disease in rats. The results showed that *T. cruzi* infected melatonin treated rats had lower levels of TBARS and also reduced NO production compared with untreated controls [105]. However, the parasite burden was not determined in this study and it is possible that melatonin might also control parasite load. The authors concluded that melatonin can protect from lipid peroxidation and oxidative stress during chronic Chagas disease. Recently, it has been proposed that melatonin could be a potent therapeutic agent to treat aged Chagasic individuals, since it possesses cooperative immunomodulatory and antioxidant effects [106]. The melatonin effects on Chagas disease are most likely due to the immunomodulator capacity, antioxidant role through the activation of NRF2, which can increase the gene expression of superoxide dismutase (SOD) and GSH, an anti-inflammatory action through the decreasing of the NF-κB pathway, which leads to the decrease of the gene expression of inflammatory cytokines.

### 4.3. Curcumin

Curcumin is a natural polyphenolic flavonoid isolated from the rhizomes of *Curcuma longa* with anti-inflammatory, immunomodulatory, and cardioprotective properties. Curcumin possesses potential to prevent and treat diverse infectious, neoplastic, cardiac and immune disorders [107,108,109]. Studies in which *T. cruzi*-infected mice were treated for 35 days with orally administered curcumin and then several parameters were determined [110]. Curcumin pretreatment inhibited fibroblast parasite invasion and the expression of low-density lipoprotein receptor, which is involved in *T. cruzi* host cell invasion. In mice it was capable of reducing parasitemia and also parasite load in the heart of curcumin-treated infected mice, though there was a decrease in the expression of antioxidative enzymes such as SOD, catalase and peroxidase [111]. Furthermore, curcumin-treated infected mice had a 100% rate survival compared to 60% rate survival of the untreated controls [110]. Studies aimed to investigate the combined effect of curcumin and BZN have demonstrated that curcumin can limit the toxic effects of BZN and enhances its antiparasitic effect. Curcumin was administrated once a day (by gavage) together with BZN for 20 days after 4 days post infection with *T. cruzi* [111] and it was observed that curcumin had limited antioxidant, anti-inflammatory and antiparasitic activities when administered alone. However, when curcumin and BZN were administered together, a drastic decrease of mortality, parasitemia, parasite load, cytokine levels, myocardial inflammation, and oxidative stress cardiac tissue damage was observed and this drastic decrease was greater than the effects of BZN alone [111]. Curcumin as a co-adjuvant also reduces the liver toxicity of BZN improving the parasite cure rate. Those results suggest that a combination of curcumin with low BZN doses might be a better therapy for Chagas disease than BZN alone [111]. In other study, Hernandez et al. [112] found that curcumin treatment of acute Chagasic mice enhanced survival and decreased the inflammatory process in the heart by blocking the Ca^+2^-dependent nuclear factor activated T cell 1 (NFATc1) transcriptional pathway, thus decreasing the secretion of endothelin-1 (ET-1) from *T. cruzi*-infected vascular endothelium cells. ET-1 is a pathogenic peptide which can cause vascular injury, platelet aggregation, cardiac remodeling, and enhanced secretion of inflammatory mediators affecting the cardiomyocytes [112]. Curcumin was also able to reduce the pro-inflammatory cytokines (IL-6, TNF-α) secretion in the infected mice [113]. Curcumin therapeutic limitation is its bioavailability; therefore, new nanomedicines have been formulated to overcome this problem. Recently, Hernandez et al. [113] reported that oral therapy of curcumin-loaded particles together with suboptimal doses of BZN was able to effectively reduce the myocardial parasite load, inflammation, fibrosis and cardiac hypertrophy in mice at the chronic phase of Chagas disease. These treatments with suboptimal doses of BZN and curcumin-loaded nanoparticles also decrease the expression of myocardial proinflammatory cytokines/chemokines, the level/activity of matrix metalloproteinases (MMP-2 and 9), and the iNOS/cyclooxygenase involved in cardiac remodeling. Taken altogether, those results suggest that Curcumin/suboptimal BZN doses might be an effective therapy to treat Chagas disease at both the acute and chronic phases. Curcumin benefits in Chagas could be exerted through the inhibition of the NF-κB pathway, which reduces the inflammatory response in the heart and by activating the NRF2 transcription factor, which leads to an activation of gene expression of enzymes involved in the antioxidant defense system.

### 4.4. Resveratrol

Resveratrol is a polyphenol secondary metabolite of plant origin and it is also found in many fruits that can control tissue damage in degenerative diseases, cancer, and has a major therapeutic role as a cardiovascular system protector to prevent heart failure of different etiologies [114]. It acts as an anti-inflammatory, antioxidant, and antimicrobial, and has inducing effects on the expression of endothelial NO synthetase (eNOS) and several antioxidant properties, exerted by activation of the antioxidant-defense gene NRF2, increased expression of mitochondrial SOD2, and inhibition of NOX2 and NOX4 expression [115]. It also has scavenging activity of superoxide, hydroxyl, and peroxyl free radicals [115]. Resveratrol treatment of chronically *T. cruzi*-infected mice for 30 days, after 60 days post infection, improved electrophysiological parameters of heart function compared with untreated infected controls [116]. The treatment with resveratrol also stabilized Mn-superoxide (SOD2) levels and decreased lipid peroxidation in the hearts of Chagasic mice. The reduced ROS levels decrease the oxidative damage in the heart and improved its function and also reduced parasite burden as a consequence of curcumin treatment. Recently, a study designed to study the effects of resveratrol on the liver of mice with acute Chagas disease has shown that resveratrol is capable of augmenting SOD and GST enzymatic activities in infected mice [117]. The resveratrol treatment was also able to reduce protein thiols in the liver of treated mice compared to infected untreated controls. Although the infection caused high levels of lipid peroxidation, NO and ROS levels in the liver tissue, resveratrol was able to minimize the negative effects of those agents during the acute phase of Chagas disease [117]. The effects of resveratrol are exerted by the activation of the antioxidant defenses through NRF2, which is able to activate mitochondrial-located SOD2 gene expression and inhibits NADPH oxidases (NOX) NOX2 and NOX4 expression. Furthermore, resveratrol activates the AMPK pathway, however, this activation is SIRT1-independent [116].

### 4.5. Astaxanthin

Astaxanthin (ASTX) is a reddish carotenoid found in many plants, microorganisms and sea animals and belongs to the xanthophyll class of compounds. It is a powerful antioxidant with anti-inflammatory activities and immunomodulatory properties. ASTX can inhibit lipid peroxidation and stabilize free radicals to decrease oxidative stress damage, thus protecting biologically important enzymes. This molecule can also counteract oxidative stress caused in some diseases in the heart preventing tissue damage and heart failure [117]. We have found only one study evaluating the effect of ASTX during the acute phase of Chagas disease in mice. In this study it was observed that ASTX does not have beneficial effects during acute infection, either alone or in combination with NFX, since it did not control parasitemia and increased heart lymphoplasmacytic infiltration [118]. Furthermore, ASTX showed a negative effect in infected mice cotreated with NFX. However, it remains to be investigated whether ASTX could have a positive effect in combination with low BZN doses. Another antioxidant that has been studied in the acute phase of Chagas disease is the B-blocker carvedilol. However, as well as astaxanthin, it did not show any benefits, since it does not reduce the parasitemia and augment the inflammatory infiltration in the treated mice [119].

### 4.6. Phenyl-alfa-tert-butyl Nitrone

Phenyl-alpha-tert-butyl Nitrone (PBN) is a nitrone-based antioxidant, which can scavenge free radicals and also inhibits free radical generation. The effects of PBN in rats during the acute and chronic phases of Chagas have been studied by orally administrating the antioxidant during three weeks together with BZN [120]. The combined treatment was able to decrease ROS levels, oxidative adducts, hypertrophic gene expression, collagen deposition and preserve the respiratory chain efficiency and energy status in the hearts of chronically infected mice compared to those treated with BZN alone. Similarly, in a study in which mice at the acute phase were treated with PBN (by injection), it was observed that PBN treatment reduced mitochondrial ROS production in the infected myocardium and arrested oxidative damage-induced loss of mitochondrial membrane integrity. Additionally, the PBN treatment preserved redox potential-coupled mitochondrial gene expression and improved cardiac ATP level and respiratory complex functions in the myocardium of infected mice [121]. In conclusion, PBN could be effective to reduce oxidative damage and preserve heart function in *T. cruzi*-infected individuals. An additional action of PBN might be due to the inhibition of the NF-κB transcription factor, leading to the inhibition of iNOS and inducible cyclooxygenase (COX2) gene expression, which require this transcription factor to be expressed [122]. Additionally, the inhibition of NF-κB by PBN would result in a reduced inflammatory response, since the expression of genes involved in this pathway are NF-κB-dependent [123].

### 4.7. Tempol

Mitochondria-targeted therapies have focused on selecting the mitochondrial functions of trypanosomatids, since this organelle is different from their mammalian counterparts. However, therapies designed to improve mitochondrial functions are also necessary, because mitochondrial dysfunction contributes to Chagas cardiomyopathy and also to other disorders such as nervous system disorders and heart failure. Tempol has been evaluated in several studies as an agent for mitochondria-targeted therapies. Tempol is a stable synthetic compound also known as 4-hydroxy-TEMPO that mimics SOD (SOD-Mimetic). It has been used as an antioxidant evaluated in many diseases related with mitochondrial dysfunction and oxidative stress [124], since Tempol favors the metabolism of several cellular reactive oxygen and nitrogen species, inhibits lipid peroxidation, thus reducing oxidative stress tissue damage. Vilar-Pereira et al. [116] evaluated the effect of tempol (and metformin) to validate the effects of resveratrol on the chronic Chagas disease. Those studies demonstrated that tempol mimicked the effects of resveratrol in improving heart function, diminishing lipid peroxidation, but it has no effect on parasite burden. Metformin had similar effects as resveratrol and tempol [116]. Metformin is an indirect activator of the AMPK-pathway and has similar effects as resveratrol [116].

### 4.8. Desferrioxamine

Desferrioxamine (DFX) is a potent iron chelator that binds to iron in 1:1 stoichiometric to produce a stable ferrioxiamine and it has been used to study the effect of iron depletion in living beings. It also modulates inflammation, acts as a potent antioxidant under normal and oxidative stress conditions, since it decreases formation of free hydroxyl radicals and increases antioxidant enzyme levels such as Glutathione peroxidase (GPx) and SOD [125,126]. DFX has been also used to treat viral, bacterial and protozoan infections. In a study of the effect of DFX in chronic Chagas disease in mice it was observed that DFX-treated infected mice had lower levels of iron in liver compared with DFX-treated non-infected mice [127,128]. Furthermore, several mice from the DFX-treated infected mice showed negative PCR for *T. cruzi*. This is evidence that decreasing the iron in the host could lead to control of parasite load in the chronic phase of Chagas disease [127,128]. In an acute model of Chagas disease, Francisco et al. [129] reported that treatment of DFX combined with BZN reduces mortality to 0%, though BZN treatment with or without DFX also reduces mortality of *T. cruzi*-infected mice. The DFX treatment increased the levels of TBARS both in liver and serum of infected mice and also the levels of nitrate/nitrate were increased in those mice [129]. The SOD activity was augmented in DFX-treated-infected mice, however, Garg and colleagues [130] have noticed that SOD activity is increased in the heart and liver of *T. cruzi*-infected mice at the acute phase (8–21 days) of Chagas disease and after the antioxidant capacity drops. Therefore, the observed increased SOD activity might not be dependent on DFX. The effect DFX has not been evaluated in the chronic phase of Chagas disease yet.

### 4.9. Honokiol

Honokiol (HKL) is a lignan bi-phenol derived from Magnolia plants, which acts facilitating mitochondrial respiration and acts also as an antioxidant and anti-inflammatory in the myocardium. Recently, a study on the effect of HKL in chronic Chagas disease has shown that treatment with this compound can attenuate the oxidative stress-dependent heart dysfunction characteristic of chronic Chagas disease [131]. It blocks ROS-production in *T. cruzi* cultured cardiomyocytes and most importantly, HKL was able to decrease cardiac dysfunction, inflammatory tissue damage, parasitism and fibrosis in the heart of chronically *T. cruzi*-infected mice. HKL also decreases ROS levels and oxidative-nitrosative stress in the heart of mice in the chronic phase of Chagas disease. It was concluded that HKL might be a useful antioxidant to prevent the long-term evolution of Chagas heart disease, since it provides benefits preventing oxidative stress-induced mitochondrial dysfunction and the impairment of antioxidant defenses. Honokiol can activate the AMPK/NRF2/SIRT3 pathway, leading to increased antioxidant defense and perhaps also an inhibition of the inflammatory response [131]. SIRT3 is the main mitochondria-located sirtuin, which regulates by deacetylation key enzymes of OXPHOS, therefore, controls energy metabolism in the cardiomyocytes.

### 4.10. Phytochemical Compounds

*Morus nigra* is popularly known as mulberry or blackberry. Leaf extracts from this plant contain several chemical compounds such as flavonoids, triterpenes, steroids. and alkaloids. The antioxidant potential of *M. nigra* leaf extracts, especially phenolic compounds (flavonoids), was evaluated in chronically *T. cruzi*-infected mice. There was an important action of *M. nigra* extracts, since they can reduce the parasitemia at the acute phase of Chagas disease, however, at the chronic phase they have activity on some antioxidant defenses and minimized the tissue inflammatory process [132]. The authors concluded that *M. nigra* leaf extracts have important actions on Chagas disease progression. Recently, a study has evaluated the trypanocidal effects of ethanolic extracts from *C. fimbriata* and concluded that in addition to the trypanocidal effects on *T. cruzi*-infected cells, those extracts might have the capacity to boost the immune system [133]. However, those extracts have not been in vivo evaluated and more research is needed to know the beneficial effects of those extracts as antioxidants to treat Chagas disease progression.

### 4.11. Apocynin

Apocynin is a natural product methoxy-substituted catechol, which is able to inhibit the assembly of NADPH-oxidase subunits (NOX2), an enzyme produced by activated phagocytes, which is responsible for ROS production [134]. Apocynin is an anti-inflammatory agent, although its mode of action is not well defined yet, since some authors consider it as an antioxidant, because it is unable to inhibit the vascular NADPH oxidases and acts through another pathway to inhibit ROS production [135]. Regarding its action mechanism, Dhiman and Garg have studied the effect of apocynin on *T. cruzi*-induced myocarditis during the acute and chronic phases of Chagas disease [136]. Apocynin treatment of *T. cruzi*-infected mice at the acute phase showed a significant decline in ROS/NOX production, decreased proinflammatory cytokine production, a decline in splenic phagocyte proliferation and decreased splenic T-cell proliferation. However, the parasite burden was increased in apocynin treated infected mice, though inflammatory cell infiltrate, acute myocarditis, and tissue oxidative adducts were decreased in those mice. At the chronic phase, heart hypertrophy due to increased cardiomyocytes size and heart fibrosis were controlled in apocynin-treated Chagasic mice [136]. Those results suggest that apocynin could control Chagas disease progression and the heart failure produced at the chronic phase. Considering the anti-inflammatory activities of apocynin, we might conclude that this compound definitely deserves further study.

## 5. Therapeutic Uses of Antioxidants and Anti-Inflammatories for Chagas Disease Myocarditis

Cardiomyopathy is one of the most common and serious manifestations of chronic Chagas disease. It has been suggested that cardiomyopathy is the result of the progressive tissue damage caused for the continuous oxidative stress, instead of a directed continued action of *T. cruzi* [92]. The continuous oxidative stress triggers the immune response that causes tissue damage and the mitochondrial dysfunction in the cardiomyocytes that produces elevated ROS levels. Therefore, antioxidants have emerged as a promising therapeutic approach to treat the progressive tissue damage in Chagas disease. During treatment with antioxidants, several oxidative markers are evaluated in the serum and red blood cells of the patients. Typical evaluated markers are TBARS, carbonyl protein (PCN), glutathione reductase (GR), superoxide dismutase (SOD), reduced glutathione (GSH), glutathione peroxidase (GPx), nitric oxide (NO), as well as inflammatory markers in the heart of Chagasic patients.

A summary of the evidence that supports the use of antioxidants in experimental Chagas disease can be found in Table 1 from the excellent review by Sanchez-Villamil et al. [137]. On the other hand, evidence of the use of antioxidant therapy in human patients is described in Tables 1–4 in a systematic review by Freitas et al. [138]. Those antioxidant treatments were done in combination or not with the anti-Chagasic drug BZN. Freitas et al. [138] also included the treatment with anti-inflammatory agents. In summary, we can conclude that most of therapeutic treatments with antioxidants in experimental Chagas disease do not seem to have total efficacy in the outcome of the disease. Even though some of the treatments decreased oxidative markers at the same time, others were increased. Similar results were obtained in the studies described by Freitas et al. [138], although they concluded that vitamin supplementation is a promising antioxidant therapy together with an anti-inflammatory treatment. However, we cannot make a definitive conclusion, since it is most likely that the treatment protocol and the antioxidant dose must be adjusted to obtain optimal results. Table 1, in this article, presents a summary of the effect of the antioxidant treatment on different oxidative markers in experimental Chagas disease. In Table 2 the main clinical trials carried out with antioxidants on Chagas disease patients and the effect on several oxidative markers are presented. In Table 3 a summary with the studies that have been carried out with antioxidants as co-adjuvant therapy in experimental Chagas disease is presented. All the ongoing clinical trials with antioxidant therapy in Chagas disease patients can be consulted in the review by Freitas et al. [138].

## 6. Concluding Remarks

Chagas disease is one of the most prevalent neglected tropical diseases in Latin America and causes the death of several thousand people each year. Despite of decades of research, an effective treatment has not been found yet and BZN is the only available drug for treatment. This drug is trypanocidal and can be effective at the acute phase of the disease, however, once the tissue damage is established, the disease can progress independently of the parasite presence. Mitochondrial ROS produced by the infected cardiomyocytes triggers most of the tissue damage and leads to a mitochondrial dysfunction. Until new drugs become available, therapies should focus on preserving cardiomyocyte function to avoid heart failure, since myocarditis is one of the most severe features of chronic Chagas disease. Antioxidant co-adjuvant treatment is a promising therapy for both at the acute and chronic phases of the disease and has worked successfully in experimental models of Chagas disease, however, it seems far from application to patients. Most clinical trials with antioxidants have not been as effective as expected, although some antioxidants have shown some benefits in treated Chagasic patients. It must be noted that many of the antioxidants have been tested only once and different doses or treatment time periods have not been evaluated. Usually markers are evaluated, but health quality or extension of the patient life span, compared with patients without treatment, are not mentioned in the results of clinical trials. Additionally, since many antioxidants have been evaluated only once, the results have not been reproduced by other clinicians. Special interest should be put on antioxidants that can promote mitochondrial biogenesis through the PGC-1α coactivator, stimulation of gene expression through the NFR2 transcription factor, increase the gene expression of antioxidant enzymes, decreasing the proinflammatory cytokine response and blocking or scavenging ROS and RSN, which are responsible for most of the tissue damage. In addition, the action mechanisms of the antioxidants must be studied in detail in order to stimulate different pathways in the damaged cell in order to preserve its function. We expect that in this decade new tools to fight Chagas disease will became available.

## Figures and Tables

**Table 1:** Oxidative markers and antioxidants evaluated in *T. cruzi* infection and Chagas disease.

Antioxidant	Oxidative Stress Marker						
	Mitochondrial Function	NO Production	Lipid Peroxidation	PCN	GTN	SOD	CAT
ASTX Apocynin			X				
			*	*			
Carvedilol			NS	X		Xa, *(−h)	X
Curcumin DFX Flavonoids			*	*(+)		*(−)	*(−)
		X	X			*(+)	
			X				
HKL	*					*(+)	*(+)
Melatonin		X	*				
PBN	*						
Resveratrol			*			*(−)	
Tampol	*		*				
Vitamin C or E			X			X	X
Vitamin C/E			NS		*		

*: Studies show significant decrease of the oxidative stress marker. X: Studies show significant detrimental results of the oxidative stress markers. NS: Studies show non-significative changes in the oxidative stress marker. *(−): Studies show significant decrease in enzymatic activity. *(+): Studies show significant increase in enzymatic activity. Studies in animal models are indicated as “a” and clinical studies are indicated as “h”. ASTX: astaxanthin; DFX: deferoxamine; NO: nitric oxide; PCN: protein carbonylation; GTN: glutathione; SOD: superoxide dismutase; CAT: catalase.

**Table 2:** Main results of antioxidants therapies in patients with Chagas cardiomyopathy.

Study	Design	Intervention	Results
Barbosa et al. 2016	Prospective, open cohort	BZN (2 months) and vitamins C and E supplementation (6 months)	Reduction of PVC episodes in patients with severe Chagasic cardiopathy. Reduction of serum markers of oxidative stress. No significant reduction of PVC in patients with lower degree of cardiac damage.
Budni et al. 2013	Prospective, open cohort	Carvedilol (6 months) and 6 months wash out vitamins C and E (6 months)	Reduced oxidative stress evidenced by decreased markers such as SOD, TBARS, NO, GPx, GR, CAT, ADA, PC. Reduction was most evident when Carvedilol was associated with antioxidant vitamins.
Ribeiro et al. 2010	Prospective, cohort	BZN (2 months) and vitamins C and E supplementation (6 months)	SOD, CAT, GPx and PC activities were reduced, and vitamin E level was reduced after BZN treatment. SOD, GPx, and GR activities were reduced and PC, TBARS, NO, and GSH levels were reduced after vitamin supplementation.
Maçao et al. 2007	Prospective, cohort	Vitamins C and E supplementation (6 months)	Reduction of plasma levels of TBARS and PC and increased GSH content in erythrocytes in group I. Lower plasma levels of vitamin E in patients with most severe disease. Reduction of myeloperoxidase and GST activities in groups II, III, and IV. Increase of GR and GPx activities in group I. Increase of CAT activity in group II. Increase of NO activity in groups II and III.

BZN: benznidazole; PVC: premature ventricular contraction; SOD: superoxide dismutase; TBARS: thiobarbituric acid-reactive substances; NO: nitric oxide; GPx: glutathione peroxidase; GR: glutathione reductase; CAT: catalase; ADA: adenosine deaminase; PC: protein carbonyl; GSH: glutathione reduced.

**Table 3:** Antioxidant treatment in Chagasic animal models.

Model	Age	*T. cruzi* Strain and Dose	Treatment	Antioxidant/ Oxidant Stress Marker	Tissue	Reference
C57BL/6 mice	6–8 weeks	Sylvio × 10 (1 × 10^4^)	50 mg/kg PBN (i.p.) on alternate days for 3 weeks	Respiratory complex activities, MDA, GSH, ATP, H_2_O_2_	Heart, heart mitochondria	[121]
SWRJ/W male mice	4 weeks	Y (1 × 10^2^)	5 mg/50 μL/day desferrioxamine (i.p.) 14 days prior to infection and for 21 days i.p.	GSH, TBARS, PCN, nitrate/nitrite	Serum, liver	[129]
Sprague Dawley rats	4–5 weeks	Sylvio × 10 (1 × 10^4^)	1.3 mM PBN and/or 0.7 mM benznidazole for three weeks in drinking water	ROS, TBARS	Heart, heart mitochondria	[120]
Sprague Dawley rats	4–5 weeks	Sylvio × 10 (1 × 10^4^)	1.3 mM PBN and/or 0.7 mM benznidazole for three weeks in drinking water	PCN	Heart, heart mitochondria	[138]
CD1 mice	6–8 weeks	Brazil (5 × 10^4^)	100 mg/kg/day curcumin for 35 days orally	mRNA levels of proteins/enzymes	Heart	[110]
SWR/J male mice	3 weeks	QM1 (5 × 10^4^)	10 μL vitamin C (D50 mg or D500 mg) per day for 60 days or 180 days orally	TBARS, total peroxide, GSH	Plasma, heart, colon, skeletal muscle	[99]
Wistar male rats	NR	Y (1 × 10^5^)	5 mg/kg melatonin/day for 60 days orally	Nitrite production in macrophages, TBARS in plasma	Plasma, spleen	[138]
SWR/J female mice	8–12 weeks	Y (2 × 10^3^)	Curcumin (C) +/− Benznidazole (B) for 20 days by gavage. C100 (+/−B50–B100), B50–B100 only (mg/kg/day)	MDA, PCN		[111]
BALB/c male and female mice	5–7 weeks	Colombian (2 × 10^2^)	15 mg/kg trans-resveratrol (i.p.) or 40 mg/kg resveratrol, 500 mg/kg metformin, 100 mg/kg/Tempol or 25 mg/kg benznidazole for 30 days orally	TBARS		[116]
Swiss SWR/J male mice	52 weeks	Y (2 × 10^3^)	500 mg/day vitamin C/800 UI/day vitamin E for 15 days orally	TBARS, catalase, PCN, GST and SOD activities, nitrite/nitrate, 8-OHdG		[102]
Swiss SWR/J male mice	3 weeks	QM2 (5 × 10^4^)	500 mg/day vitamin C/800 UI/day vitamin E (individually and in combination) for 60 or 120 days	FRAPS, GSH, TBARS		[101]
Swiss SWR/J male mice	3 weeks	QM2 (5 × 10^4^)	20% blackberry plant extract (25–75 μL/day) for 180 days orally	TBARS, FRAPS, GSH, sulfhydryl groups		[132]
BALB/c female mice	4–6 weeks	Ninoa (10)	10 mg/kg/day astaxanthin +/− 10 mg/kg/day nifurtimox for 60 days orally	MDA		[138]
Swiss SWR/J male mice	6 weeks	Y (1 × 10^4^)	7.14 mg/kg/day vitamin C +/− 100 mg/kg benznidazole for 15 days by gavage	TBARS, ROS		[97]
Swiss SWR/J male mice	3.5 weeks	QM2 (5 × 10^4^)	500 mg/day vitamin C for 60 days in drinking water	FRAPS, GSH, GST, plasma sulfhydryl group, nitrate/nitrite		[100]
C57BL/6 male mice	8–10 weeks	Colombian (50)	25 mg/kg/day carvedilol +/− 100 mg/kg/day benznidazole for 23 days by gavage	SOD and CAT activities, TBARS, protein carbonyls		[119]
C57BL/6 male and female mice	6–8 weeks	Brazil (1 × 10^4^)	0.2 mg/kg/day honokiol or 100 mg/kg/day benzdidazole for 57 and 85 days	CAT, SOD activities, ROS, MDA		[131]
C3H/HeN male mice	6–8 weeks	Sylvio ×10 (1 × 10^4^)	1.5 mM apocynin for 150 days in drinking water	ROS		[136]

BZN: benznidazole; PVC: premature ventricular contraction; SOD: superoxide dismutase; TBARS: thiobarbituric acid-reactive substances; NO: nitric oxide; GPx: glutathione peroxidase; GR: glutathione reductase; CAT: catalase; ADA: adenosine deaminase; PC: protein carbonyl; GSH: glutathione reduced.
